# Dynamic Lactate Level: An Effective Predictor of Short-Term Mortality After Type A Aortic Dissection Surgery

**DOI:** 10.31083/RCM50931

**Published:** 2026-06-29

**Authors:** Zhiyan Chen, Yanchao Qi, Ding Yu, Minjia Zhu, Ying Lv, Yang You, Hongzhao Li, Yue Shi, Jun Liu

**Affiliations:** ^1^Heart Center, The First Hospital of Hebei Medical University, 050000 Shijiazhuang, Hebei, China; ^2^Intensive Care Unit, Cardiac and Panvascular Center, People’s Hospital of Xinjiang Uygur Autonomous Region, 830001 Urumqi, Xinjiang Uygur Autonomous Region, China; ^3^School of Disaster and Emergency Medicine, Tianjin University, 300072 Tianjin, China

**Keywords:** aortic dissection, lactate, lactate clearance, risk factors, short-term mortality

## Abstract

**Background:**

Type A aortic dissection (TAAD) is a life-threatening cardiovascular emergency frequently accompanied by elevated lactate (Lac) levels during surgery. This study investigated the relationship between early postoperative changes in Lac levels and short-term mortality in patients with TAAD.

**Methods:**

A retrospective analysis was conducted on TAAD patients who underwent surgery at our institution. Lac levels were measured immediately after surgery and on postoperative days (PODs) 1 and 3. Logistic regression was used to analyze patient demographics and clinical outcomes, and to identify short-term mortality risk factors. Receiver operating characteristic (ROC) curves were constructed to assess the association between Lac levels, lactate clearance (LC), and short-term mortality.

**Results:**

A total of 146 patients were included; 101 survived, and 45 died. Non-survivors had significantly higher Lac levels at intensive care unit (ICU) admission and on PODs 1 and 3. Time-dependent multivariate Cox regression analysis revealed that the trend in Lac levels during the first 3 days in the ICU was an independent risk factor for short-term mortality (hazard ratio = 1.33). On POD3, the area under the curve (AUC) for predicting short-term mortality was 0.838 for Lac and 0.667 for LC. Kaplan–Meier analysis showed an increased mortality risk with Lac >1.75 mmol/L or LC ≤37.23%. Age, sex, and liver and kidney function did not significantly affect the predictive value of Lac and LC for mortality.

**Conclusion:**

Dynamic postoperative changes in Lac levels are significantly associated with adverse clinical outcomes in patients with TAAD. Lac and LC measured on POD3 may serve as reliable indicators for predicting short-term mortality in these patients.

## 1. Introduction

Type A aortic dissection (TAAD) is a cardiovascular emergency with an extremely high mortality rate due to the high incidence of postoperative complications. Surgical intervention remains the most effective treatment for TAAD. However, despite ongoing progress in surgical techniques, the perioperative mortality risk remains substantial, with a 30-day postoperative mortality rate ranging from 8% to 30% [1,2]. The prognosis of TAAD patients is determined by multiple factors, including age, the extent and location of the dissection tear, existing comorbidities, operative duration, malperfusion syndrome, and postoperative complications [3]. Therefore, the identification of robust prognostic factors is crucial for guiding individualized treatment and improving patient outcomes.

Organ malperfusion is one of the major life-threatening complications of TAAD, affecting approximately 33% of patients [4]. Almost 15% of TAAD patients present with lower limb ischemia [5]. Lactate (Lac) is the final product of anaerobic metabolism and functions as an objective biomarker in arterial blood and as an indicator of tissue perfusion status [6,7]. Previous studies have established that elevated Lac levels are associated with mortality in cardiovascular diseases [6,8]. Additionally, their prognostic significance in TAAD has also been demonstrated [9]. However, current research has predominantly focused on single time-point Lac measurements [10], and the predictive role of dynamic Lac changes for short-term postoperative mortality in TAAD patients remains insufficiently explored. Therefore, the aim of the present study was to investigate the predictive value of dynamic postoperative Lac changes for short-term mortality in TAAD patients. We also determined the optimal Lac cut-off value, thereby providing an objective basis for early clinical risk stratification.

## 2. Materials and Methods

### 2.1 Study Design and Population

**Fig. 1.**
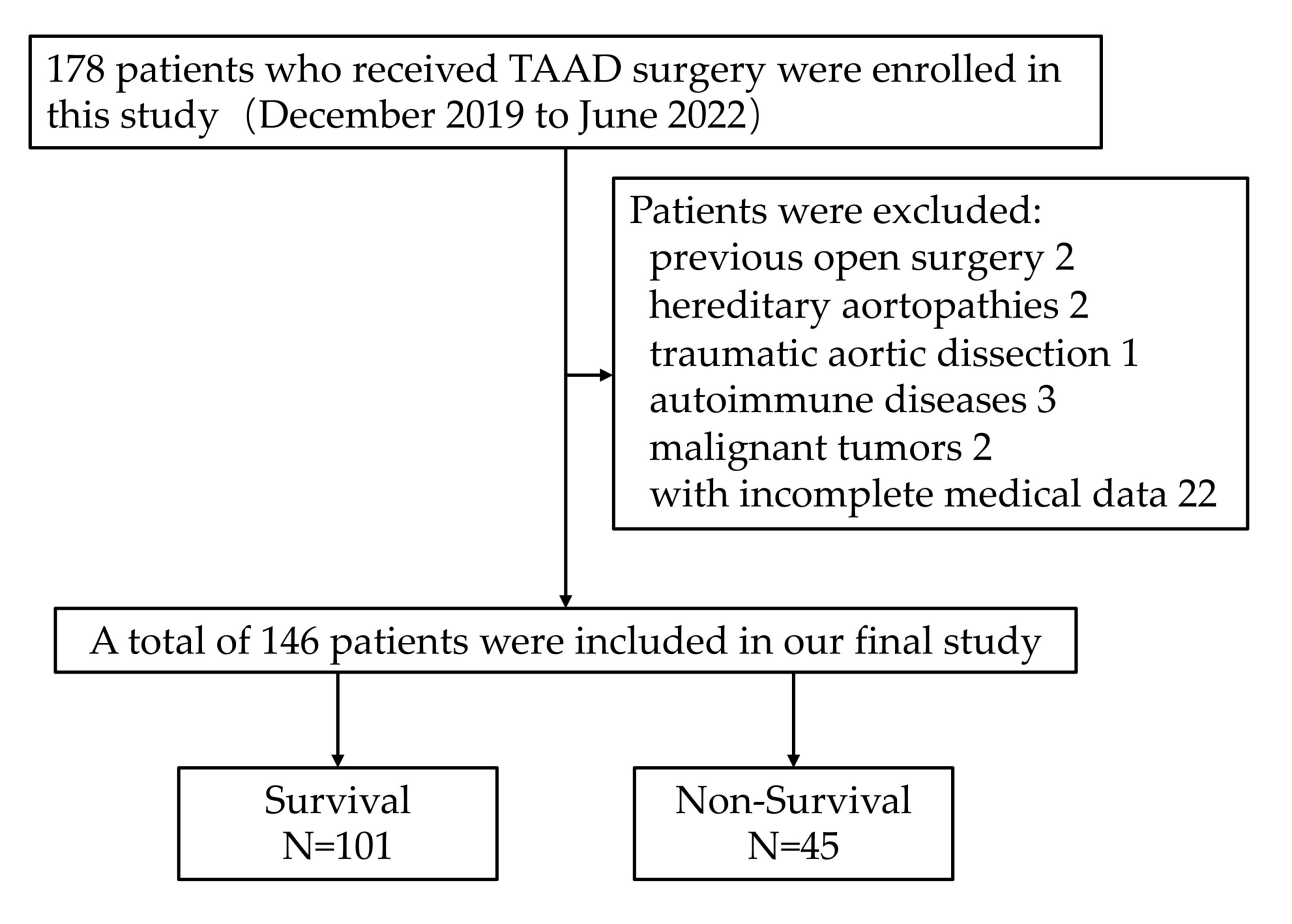
**Flowchart of the study population**. TAAD, type A aortic dissection.

This single-center retrospective study involved TAAD patients who underwent surgery at the First Hospital of Hebei Medical University between 2019 and 2022 (Fig. 1). Data privacy adhered to national laws, and the study complied with the Declaration of Helsinki and local guidelines for retrospective research. The study was approved by the hospital’s ethics committee (approval No. 20220946), and the requirement for informed consent was waived due to its retrospective nature.

All patients underwent preoperative computed tomography angiography (CTA) of the aorta using a 64-slice or higher multidetector computed tomography (CT) scanner. Images were analyzed on specialized workstations to assess aortic morphology, imaging features, and Stanford classification. Inclusion criteria were: TAAD confirmed by aortic CTA, first aortic dissection episode, symptom onset to hospital presentation <14 days, no prior surgery, and age >18 years. Exclusion criteria included previous aortic surgery, hereditary aortic conditions (e.g., Marfan syndrome), traumatic or pregnancy-related dissections, incomplete medical records, and any autoimmune, inflammatory, infectious, hematologic, or malignant disease. After screening, 146 eligible individuals were enrolled and categorized into survival and non-survival groups based on outcomes.

### 2.2 Data Collection and Definitions

Clinical data were extracted from the hospital’s electronic medical records and included information on demographics, cardiovascular risk factors, clinical presentation, anatomical details, treatments, surgical specifics, and hospitalization. Patient information included sex, age, body mass index (BMI), and medical history. Surgery details encompassed type, cardiopulmonary bypass, and postoperative complications. Intensive care unit (ICU) data included mechanical ventilation duration and length of ICU stay. Laboratory data included routine blood tests, liver and kidney function, coagulation, and arterial blood gas analysis. In addition, serial measurements were made at three time points, similar to the approach used for Lac (see below). In subgroup analyses, abnormal liver function was broadly defined as alanine aminotransferase (ALT) activity >50 U/L, based on the upper limit of the laboratory reference range. Abnormal renal function was defined as creatinine (Cr) >98 µmol/L. Because Cr levels are susceptible to individual variation, this threshold was derived from the average of the upper limits of the laboratory reference ranges for both males and females. A comprehensive bedside assessment of cardiac structure and function was conducted by skilled echocardiographers utilizing a standardized ultrasound system (Philips EPIQ 7C, Philips Healthcare, Andover, MA, USA). The parameters measured included aortic diameter (AD), left atrial transverse diameter (LATD), left ventricular fractional shortening (LVFS), and left ventricular ejection fraction (LVEF), among others. The primary focus of this TAAD surgery patient cohort was in-hospital short-term mortality.

### 2.3 Lac Measurement

Arterial blood Lac levels were routinely measured at different postoperative time points using the blood gas analyzer (ABL90 FLEX, Radiometer Medical ApS, Brønshøj, Denmark): immediately postoperative (IPO) upon ICU admission, postoperative day 1 (POD1), and postoperative day 3 (POD3). Lactate clearance (LC) was defined as the percentage reduction in Lac from baseline to time t: LC_POD1_ = (Lac_IPO_ – Lac_POD1_) ÷ Lac_IPO_ × 100%; LC_POD3_ = (Lac_IPO_ – Lac_POD3_) ÷ Lac_IPO_ × 100%. Here, Lac_IPO_ is the baseline Lac level, while Lac_POD1_ and Lac_POD3_ are the Lac levels on POD1 and POD3, respectively.

### 2.4 Statistical Analysis

Data analysis was conducted using R software (v4.5.1, R Core Team, Vienna, Austria). Continuous variables were assessed for normality with the Kolmogorov-Smirnov test. Normally distributed data are shown as the mean ± SD and compared using the *t*-test, while non-normally distributed data are shown as the median (interquartile range, IQR) and compared using the Mann-Whitney U test. Categorical variables are presented as the frequency (percentage) and compared using the χ^2^ or Fisher’s exact test. Univariate analysis was performed on all baseline characteristics. Univariate time-dependent Cox regression was used for multi-time-point indicators, and variables with *p* < 0.05 and a variance inflation factor <5 were included in multivariate analysis. Results are reported as the hazard ratio (HR) with 95% confidence interval (CI). Generalized estimating equations (GEE) analyzed indicator differences between survival status groups over time, using Type III Wald χ^2^ tests to evaluate the significance of each main effect. Receiver operating characteristic (ROC) curves were used to assess the predictive ability for short-term mortality, with calculation of the area under the curve (AUC) and the optimal cut-off value. Subgroup analyses were performed to assess the reliability of Lac and LC in predicting short-term mortality, with interaction p-values provided. Statistical significance was set at *p* < 0.05.

## 3. Results

### 3.1 Patient Characteristics

This study analyzed 146 TAAD patients who underwent surgical treatment. Comparison between the survival group (n = 101) and the non-survival group (n = 45) revealed significant differences across multiple preoperative, intraoperative, and postoperative factors, as shown in Table 1. The non-survival group was older (57.03 ± 11.84 years vs. 52.84 ± 11.49 years, *p* = 0.046) and had significantly longer operative time (9.00 [8.00, 11.00] h vs. 8.00 [8.00, 9.00] h, *p* < 0.001), cardiopulmonary bypass (CPB) time (221.50 [191.75, 278.75] min vs. 185.00 [170.50, 203.25] min, *p* < 0.001), and aortic cross-clamp (ACC) time (147.50 [133.00, 201.75] min vs. 132.00 [123.00, 145.00] min, *p* < 0.001) compared with the survival group. Furthermore, a higher proportion of patients in the non-survival group underwent the Bentall procedure (28.89% vs. 14.85%, *p* = 0.047) and coronary artery bypass grafting (20.00% vs. 7.92%, *p* = 0.036), whereas fewer underwent temporary pacemaker implantation (24.44% vs. 42.57%, *p* = 0.036). The incidence of postoperative complications was significantly higher in the non-survival group (*p* < 0.05), as detailed in Table 2.

**Table 1.** **Comparison of baseline characteristics**.

Variable	Survival Group (n = 101)	Non-Survival Group (n = 45)	*p*
Male, n (%)	70 (69.31)	32 (71.11)	0.826
Age (years)	52.84 ± 11.49	57.03 ± 11.84	0.046
HR at admission (bpm)	79.00 [65.00, 90.00]	74.00 [65.00, 85.00]	0.406
SBP at admission (mmHg)	142.55 ﻿± 31.31	137.68 ﻿± 33.25	0.394
DBP at admission (mmHg)	77.62 ± 18.15	76.57 ± 17.59	0.743
BMI	25.95 [23.44, 29.64]	26.67 [24.49, 29.39]	0.417
Hypertension, n (%)	76 (75.25)	38 (84.44)	0.215
Diabetes, n (%)	6 (5.94)	3 (6.67)	1.000
Coronary Heart Disease, n (%)	8 (7.92)	5 (11.11)	0.540
Valvular Heart Disease, n (%)	7 (6.93)	2 (4.44)	0.722
Cerebrovascular Disease, n (%)	5 (4.95)	5 (11.11)	0.285
COPD, n (%)	1 (0.99)	0 (0.00)	1.000
Renal Dysfunction, n (%)	2 (1.98)	0 (0.00)	1.000
Hyperlipidemia, n (%)	4 (3.96)	1 (2.22)	1.000
Smoking, n (%)	37 (36.63)	21 (46.67)	0.253
Symptom-to-Surgery Time (h)	11.00 [8.00, 22.00]	10.00 [8.00, 22.00]	0.616
Surgery Duration (h)	8.00 [8.00, 9.00]	9.00 [8.00, 11.00]	<0.001
CPB Time (min)	185.00 [170.50, 203.25]	221.50 [191.75, 278.75]	<0.001
ACC Time (min)	132.00 [123.00, 145.00]	147.50 [133.00, 201.75]	<0.001
DHCA (min)	4.00 [3.00, 6.00]	5.00 [3.00, 9.00]	0.141
Sun’s Procedure, n (%)	90 (89.11)	41 (91.11)	1.000
David Procedure, n (%)	0 (0.00)	2 (4.44)	0.094
Bentall Procedure, n (%)	15 (14.85)	13 (28.89)	0.047
Wheat Procedure, n (%)	1 (0.99)	0 (0.00)	1.000
Aortic Valvuloplasty, n (%)	39 (38.61)	18 (40.00)	0.874
Ascending Aortic Replacement, n (%)	94 (93.07)	40 (88.89)	0.515
Total Aortic Arch Replacement, n (%)	93 (92.08)	44 (97.78)	0.275
TEVAR, n (%)	92 (91.09)	44 (97.78)	0.175
CABG, n (%)	8 (7.92)	9 (20.00)	0.036
Femoral Artery Repair, n (%)	13 (12.87)	2 (4.44)	0.149
Temporary Pacing, n (%)	43 (42.57)	11 (24.44)	0.036
Hemopericardium, n (%)	57 (56.44)	27 (60.00)	0.687
ICU LOS (day)	5.00 [3.00, 7.00]	7.00 [2.00, 12.00]	0.244
MV Time (h)	30.00 [15.00, 64.00]	121.00 [27.00, 218.00]	<0.001

Data are presented as mean ± SD, number (percentage) or Median (Q1, Q3). HR, heart rate; SBP, systolic blood pressure; DBP, diastolic blood pressure; BMI, body mass index; COPD, chronic obstructive pulmonary disease; Symptom-to-Surgery Time, time from symptom onset to surgery; CPB, cardiopulmonary bypass; ACC, aortic cross-clamp; DHCA, deep hypothermic circulatory arrest; ICU LOS, intensive care unit length of stay; TEVAR, thoracic endovascular aortic repair (for descending aorta); CABG, coronary artery bypass grafting; Temporary Pacing, temporary pacemaker implantation; MV Time, mechanical ventilation time.

**Table 2.** **Characteristics of early postoperative complications**.

Variable	Survival Group (n = 101)	Non-Survival Group (n = 45)	*p*
Acute Renal Failure, n (%)	24 (23.76)	39 (86.67)	<0.001
Heart Failure, n (%)	80 (79.21)	43 (95.56)	0.012
Myocardial Infarction, n (%)	10 (9.90)	21 (46.67)	<0.001
Shock, n (%)	5 (4.95)	26 (57.78)	<0.001
Respiratory Failure, n (%)	1 (0.99)	26 (57.78)	<0.001
Abnormal Liver Function, n (%)	42 (41.58)	35 (77.78)	<0.001
MODS, n (%)	0 (0.00)	28 (62.22)	<0.001
Pericardial Effusion, n (%)	23 (22.77)	17 (37.78)	0.060

Data are presented as number (percentage). MODS, Multiple Organ Dysfunction Syndrome.

### 3.2 Risk Factors for Short-term Mortality

**Fig. 2.**
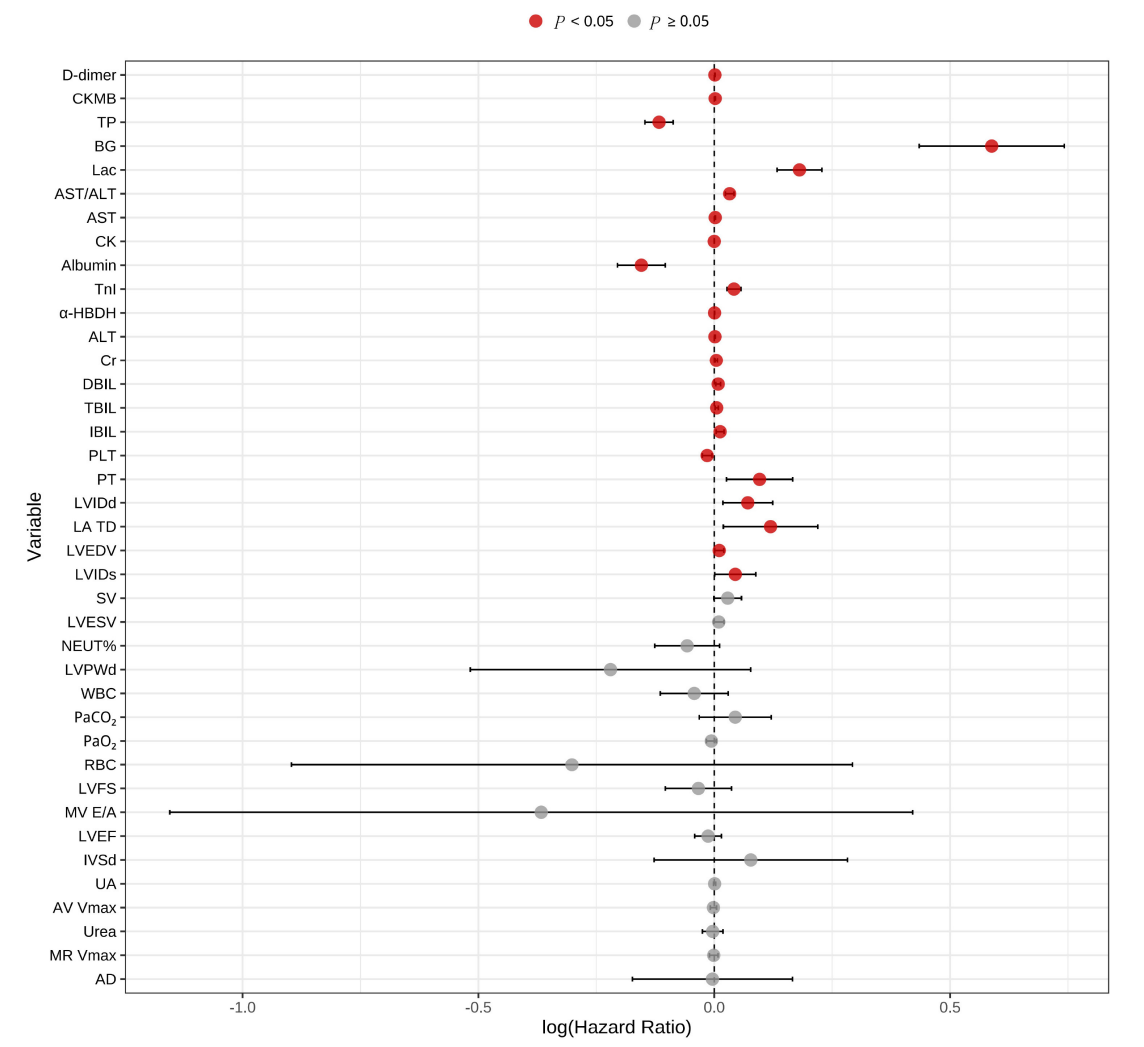
**Forest plot of univariate time-dependent cox regression for each indicator**. Red, *p* < 0.05; Gray, *p* ≥ 0.05. CKMB, creatine kinase-MB; TP, total protein; BG, blood glucose; AST, aspartate aminotransferase; AST/ALT, AST to ALT ratio; CK, creatine kinase; TnI, troponin I; α-HBDH, α-hydroxybutyrate dehydrogenase; TBIL, total bilirubin; DBIL, direct bilirubin; IBIL, indirect bilirubin; PLT, platelet; PT, prothrombin time; IVSd, interventricular septum thickness at end-diastole; LVPWd, left ventricular posterior wall thickness at end-diastole; LVIDs, left ventricular internal dimension at end-Systole; LVEDV, left ventricular end-diastolic volume; LVESV, left ventricular end-systolic volume; SV, stroke volume; MV E/A, mitral valve E/A; MR Vmax, mitral regurgitation peak velocity; AV Vmax, aortic valve peak systolic velocity; WBC, white blood cell; NEUT%, neutrophils percentage; RBC, red blood cell.

Univariate time-dependent Cox regression analysis was performed to determine the association strength between various parameters at each time point and patient outcome (Fig. 2). The results showed that 22 parameters, including D-dimer, creatine kinase-MB (CKMB), total protein (TP), blood glucose (BG), Lac, AST to ALT ratio (AST/ALT), aspartate aminotransferase (AST), creatine kinase (CK), and Albumin, were significantly associated with patient mortality (*p* < 0.05). Among these, 19 parameters showed HR values >1 (*p* < 0.05), including D-dimer, CKMB, BG, Lac, AST/ALT, AST, CK, troponin I (TNI), indirect bilirubin (IBIL), prothrombin time (PT) and LATD, indicating that elevated levels of these markers were associated with increased risk of mortality. Conversely, high levels of TP, Albumin, and PLT had HR values <1 (*p* < 0.05), indicating they may act as protective factors.

Multivariable time-dependent Cox regression analysis revealed that age (HR = 1.06, 95% CI: 1.02 –1.10, *p* = 0.003), operation duration (HR = 1.52, 95% CI: 1.18 – 1.96, *p* = 0.001), Bentall procedure (HR = 2.96, 95% CI: 1.18 – 7.38, *p* = 0.020), Lac (HR = 1.33, 95% CI: 1.20 – 1.48, *p* < 0.001), LATD (HR = 1.19, 95% CI: 1.04 – 1.37, *p* = 0.011), and AST/ALT ratio (HR = 1.03, 95% CI: 1.01 – 1.05, *p* = 0.005) were independent risk factors for short-term mortality in TAAD surgical patients (Table 3). In contrast, PLT (HR = 0.99, 95% CI: 0.97 – 1.00, *p* = 0.015) was an independent protective factor.

**Table 3.** **Multivariate time-dependent cox regression analysis**.

Variable	HR	95% CI	*p*
Lac	1.33	(1.20–1.48)	<0.001
α-HBDH	1.00	(1.00–1.00)	0.318
PLT	0.99	(0.97–1.00)	0.015
Cr	1.00	(1.00–1.01)	0.091
D-dimer	1.00	(1.00–1.00)	0.166
PT	0.97	(0.88–1.07)	0.560
TnI	1.03	(0.98–1.08)	0.196
LATD	1.19	(1.04–1.37)	0.011
AST/ALT	1.03	(1.01–1.05)	0.005
CPB Time	0.99	(0.98–1.00)	0.209
CABG	1.14	(0.42–3.15)	0.793
Surgery Duration	1.52	(1.18–1.96)	0.001
Age	1.06	(1.02–1.10)	0.003
Bentall Procedure	2.96	(1.18–7.38)	0.020
MV Time	1.00	(1.00–1.01)	0.167
Temporary Pacing	0.56	(0.27–1.17)	0.122

Cr, creatinine; LATD, left atrial transverse diameter.

Since the non-survival group had more postoperative myocardial infarctions (MI), MI was included as an adjusting variable in the Cox regression model (**Supplementary Table 1**). This analysis revealed that Lac was significantly linked to short-term mortality (HR = 1.30, 95% CI: 1.16 – 1.45,* p* < 0.001), whereas MI was not an independent predictor (HR = 1.69, 95% CI: 0.60 – 4.72, *p* = 0.319). After excluding 31 patients with postoperative MI (21.2%), analysis of the remaining 115 patients confirmed a significant association of Lac with short-term mortality (HR = 1.50, 95% CI: 1.19 – 1.90, *p* < 0.001), with a stronger effect size (**Supplementary Table 2**).

We also conducted a repeated-measures analysis employing GEE (**Supplementary Tables 3** and **4**). GEE analysis revealed a significant difference in Lac levels between the groups (Wald χ^2^ = 4.209, *p* = 0.040), with the non-survival group showing consistently higher Lac levels than the survival group. These findings align with the results of the time-dependent Cox regression analysis, indicating that in both methodological approaches, Lac was significantly associated with patient prognosis (odds ratio [OR], 1.52; 95% CI: 1.02 – 2.25, *p* = 0.040).

### 3.3 Lac and LC

**Fig. 3.**
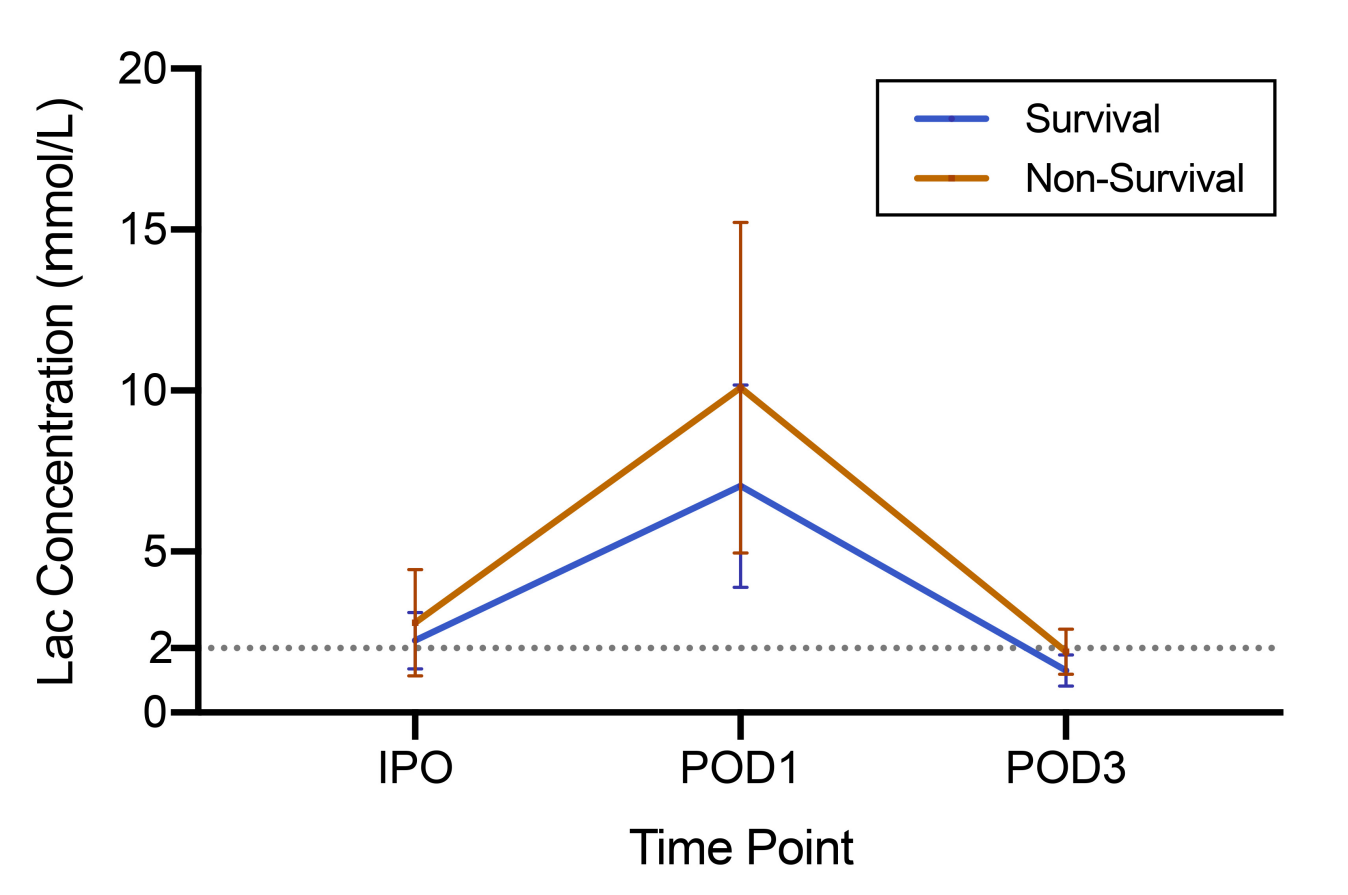
**Characteristics of Lac levels in survivors and non-survivors at different time points**. Data are presented as mean ± SD.

**Fig. 4.**
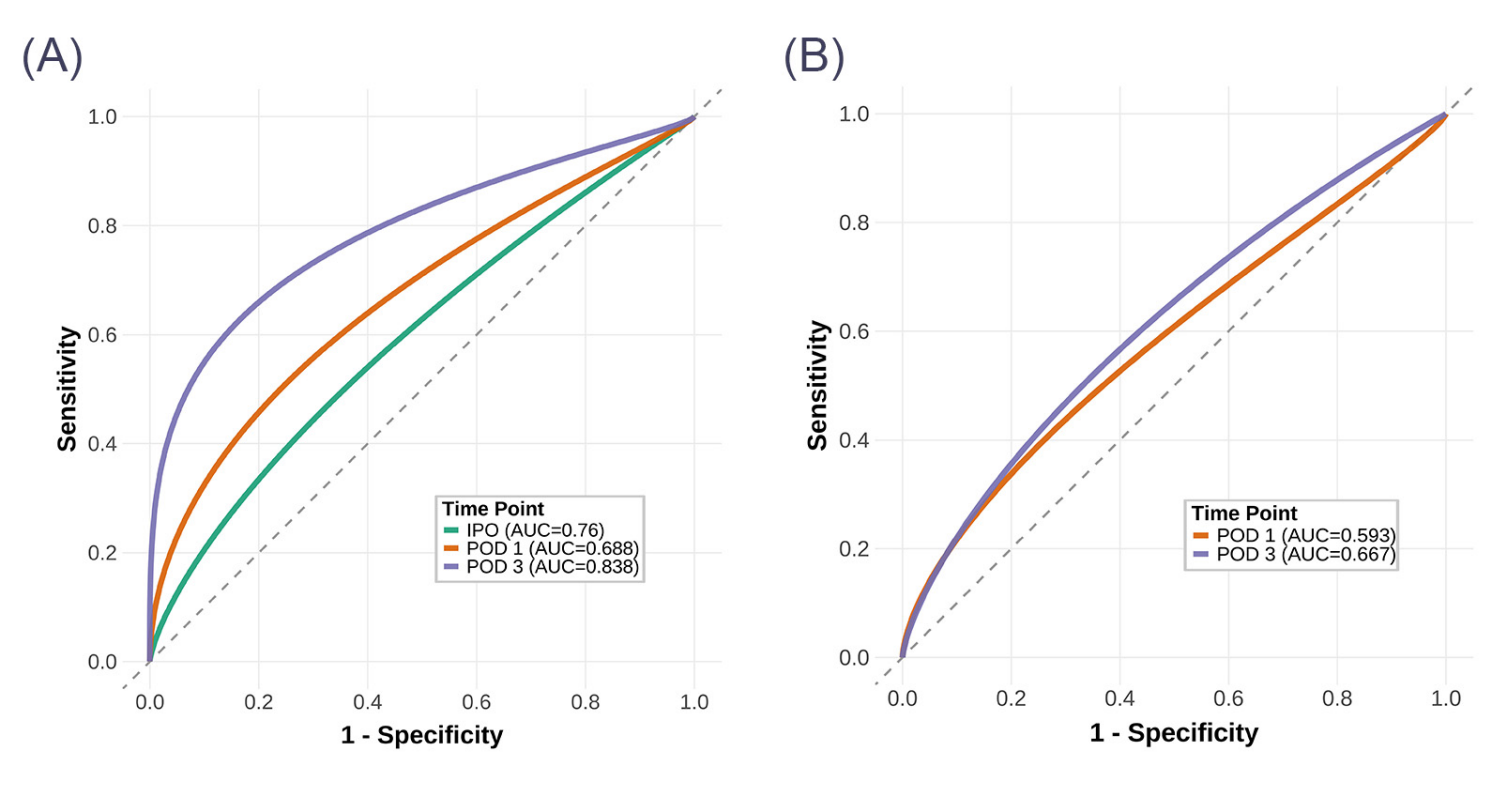
**ROC curves of Lac and LC for short-term mortality**. (A) ROC of multivariate logistic regression for association between Lac and death at IPO (Green), POD1 (Red) and POD3 (Blue). (B) ROC of multivariate logistic regression for association between LC and death at POD1 (Red) and POD3 (Blue).

Based on the temporal trends of Lac at each time point (Fig. 3), we calculated LC and constructed ROC curves to determine the predictive value of Lac for short-term postoperative mortality. As shown in Fig. 4A and Table 4, the optimal cut-off values for Lac at each time point were Lac_IPO_ = 2.15 mmol/L (AUC 0.760, 95% CI: 0.646 – 0.873, *p* < 0.001), Lac_POD1_ = 7.85 mmol/L (AUC 0.688, 95% CI: 0.584 – 0.792, *p* < 0.001), and Lac_POD3_ = 1.75 mmol/L (AUC 0.838, 95% CI: 0.758 – 0.918, *p* < 0.001). Similarly, the optimal LC cut-off values for predicting short-term mortality at the respective time points were LC_POD1_ = –218.91% and LC_POD3_ = 37.23% (Fig. 4B and Table 4), with AUCs of 0.593 (95% CI: 0.485 – 0.700, *p* = 0.090) and 0.667 (95% CI: 0.552 – 0.781, *p* = 0.004), respectively.

**Table 4.** **AUC analysis of the ROC for predicting short-term mortality**.

Time Point	AUC	95% CI	*p*	Cut-off Value	Sensitivity	Specificity
Lac_POD3_	0.838	0.758–0.918	<0.001	1.75	71.40%	87.00%
Lac_POD1_	0.688	0.584–0.792	<0.001	7.85	69.05%	72.00%
Lac_IPO_	0.760	0.646–0.873	<0.001	2.15	80.95%	91.00%
LC_POD3_	0.667	0.552–0.781	0.004	37.23	72.73%	66.34%
LC_POD1_	0.593	0.485–0.700	0.090	–218.91	62.79%	65.35%

**Fig. 5.**
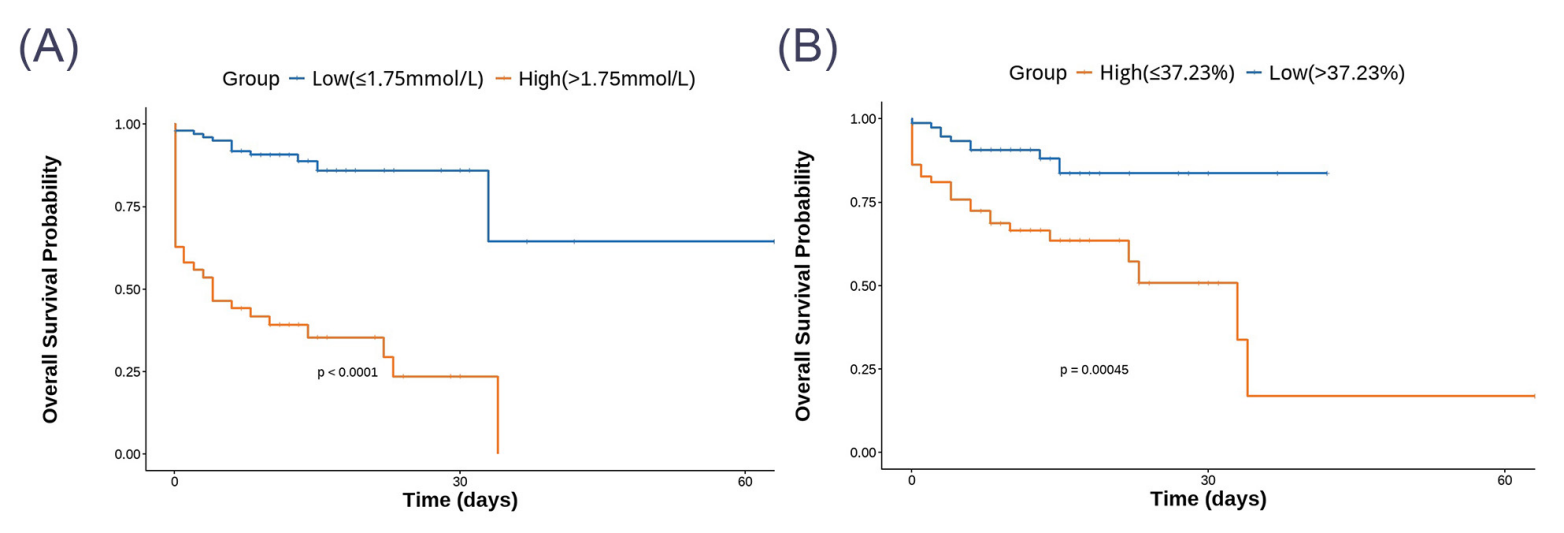
**Association between survival and Lac or LC**. (A) Probability of survival of patients with and without elevated Lac at POD3. (B) Probability of survival of patients with and without elevated LC at POD3.

Based on Lac and LC cut-off values at POD3, TAAD patients were stratified into high- and low-risk groups and Kaplan-Meier survival curves were constructed (Fig. 5). The Lac and LC cut-off values at POD3 effectively stratified patients, with survival curves for the high- (Lac >1.75 mmol/L or LC ≤37.23%) and low-risk (Lac ≤1.75 mmol/L or LC >37.23%) groups being significantly different (both *p* < 0.001).

### 3.4 Subgroup Analysis

**Fig. 6.**
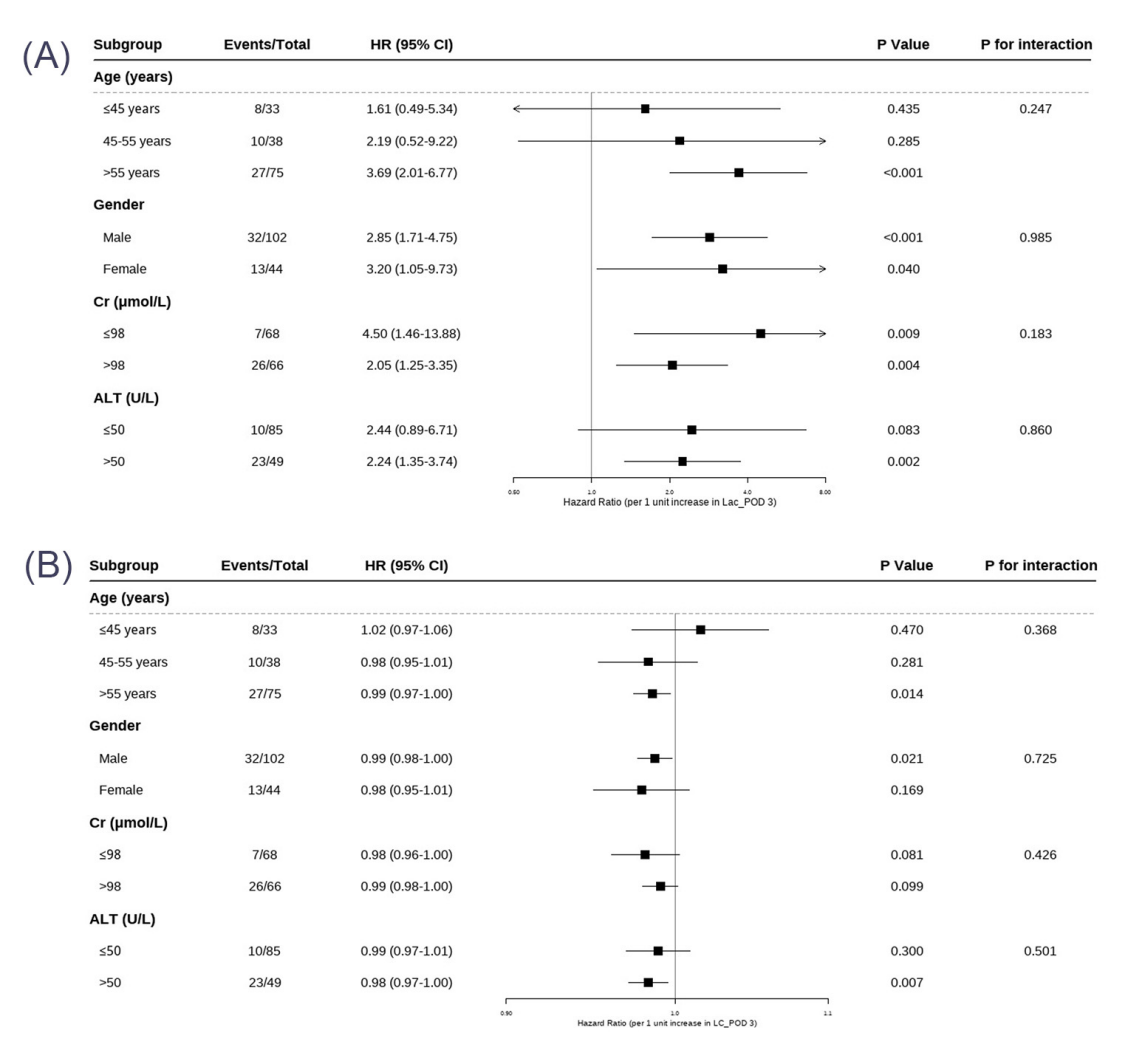
**Subgroup and interaction analyses**. (A) Forest plot showing HR and 95% CI values for LacPOD3 in predicting postoperative death in the corresponding subgroups. (B) Forest plot showing HR and 95% CI values for LCPOD3 in predicting postoperative death in the corresponding subgroups. HR, hazard ratio; CI, confidence interval.

As shown in Fig. 6A, Lac demonstrated superior predictive performance for short-term mortality in TAAD surgical patients aged >55 years compared to those aged ≤45 years (HR = 3.69, 95% CI: 2.01–6.77, *p* < 0.001), in patients with Cr ≤98 μmol/L compared to those with Cr >98 μmol/L (HR = 4.50, 95% CI: 1.45–13.88, *p* = 0.009), and in patients with ALT >50 U/L compared to those with ALT ≤50 U/L (HR = 2.24, 95% CI: 1.35–3.74, *p* = 0.002). Elevated Lac levels were consistently associated with increased mortality risk in both male and female patients, demonstrating a uniform effect direction across sexes.

LC demonstrated a superior predictive effect for reduced mortality in patients aged >50 years compared to those aged ≤45 years (HR = 0.99, 95% CI: 0.97–1.00, *p* = 0.014), a more pronounced protective effect in patients with ALT >50 U/L compared to those with ALT ≤50 U/L (HR = 0.98, 95% CI: 0.97–1.00, *p* = 0.007), and stronger predictive value in male patients compared to female patients (HR = 0.99, 95% CI: 0.98–1.00, *p* = 0.021). LC showed a protective trend regardless of renal function status, although the difference was not statistically significant (Fig. 6B).

No significant interactions were observed between age, sex, Cr, ALT, and either Lac or LC across all subgroups.

## 4. Discussion

This study focused on the predictive value of dynamic changes in Lac for adverse outcomes in patients with TAAD after surgery. The findings demonstrated a notable dynamic trend in postoperative Lac levels among TAAD patients. Specifically, elevated Lac levels at 3 days postoperatively, along with decreased LC, were independently correlated with an increased risk of short-term mortality. Dynamic Lac monitoring in the first three days postoperatively offers early risk alerts compared to single-time-point measurements. This approach provides clinicians with a dynamic, objective, and non-invasive bedside assessment tool, shifting postoperative management from static to trend-based evaluation. Improving Lac clearance could also be a therapeutic target, contributing to personalized intensive care. The incorporation of dynamic Lac monitoring into routine practice may enable timely and precise interventions, thereby improving short-term outcomes in TAAD patients.

Short-term mortality following TAAD surgery is influenced by multiple factors, including patient age, length of stay in ICU, CPB duration, and dysfunction of critical organs such as the liver and kidneys [11,12]. Consistent with previous studies, our findings indicate that non-surviving patients were generally older, experienced longer operative, CPB and ACC times, and had a higher incidence of postoperative complications (e.g., acute renal failure, heart failure, respiratory failure, MODS). CPB time is recognized as being correlated with postoperative hyperlactatemia [10,13]. Furthermore, as a potential marker for evaluating tissue perfusion, serum Lac level has shown predictive value in several studies on cardiovascular risk [14,15]. A study involving 328 TAAD patients demonstrated that individuals who developed severe acute kidney injury (AKI) postoperatively exhibited significantly elevated preoperative Lac levels. The postoperative 12-h Lac level (cut-off value: 3.3 mmol/L) was an independent predictor of AKI with 30-day mortality [15]. Puluca et al. [1] found that preoperative Lac exceeding 3.71 mmol/L was a strong predictor of early postoperative mortality in TAAD patients (OR, 7.292). The present study was focused on monitoring the dynamic trends of various indicators within the first 72 h postoperatively. We observed that Lac levels continued to rise after ICU admission and peaked, a pattern consistent with previous findings [16,17]. Importantly, our study found that dynamic changes in Lac showed strong predictive value (HR 1.33). Monitoring of trends in Lac may therefore provide earlier warning of adverse outcomes compared to a single static measurement. Consequently, we recommend dynamic monitoring of Lac, as this may improve early detection of risk and inform real-time assessment of clinical interventions.

Early postoperative elevation of the Lac level in TAAD patients is influenced by multiple factors, including hyperglycemia, prolonged CPB, liver and kidney function status, epinephrine use, and increased endogenous catecholamine secretion [10,18]. After controlling for confounding variables through multivariate analysis, our study found that the dynamic evolution of Lac within the first 3 postoperative days was an independent risk factor for short-term mortality in TAAD patients (HR 1.33). The results of the GEE analysis provided additional validation of this finding. Interestingly, Lac persisted as a significant predictor after controlling for the confounding influence of MI. Furthermore, the predictive efficacy of Lac was enhanced following the exclusion of patients with MI. These findings indicate that while MI contributes to the prognostic influence of Lac within the study cohort, it is not a primary determinant of its predictive ability. Consequently, we suggest that multi-time-point measurement of Lac can more comprehensively capture the progression of circulatory dysfunction by integrating the effects of various factors—including CPB, hemodynamic changes, and target organ perfusion—on Lac levels over time. This approach may improve predictive accuracy for the risk of early mortality. Our findings offer clinicians a dynamic, objective, non-invasive, and convenient tool for bedside assessment.

Elevated Lac in TAAD surgical patients can serve as a highly sensitive indicator for the prediction of adverse outcomes. Lin et al. [19] observed that the peak Lac level 24 h postoperatively was an effective predictor of short-term mortality (AUC 0.729). Wang et al. [16] also reported significantly increased Lac levels after ICU admission in TAAD patients, with a 24 h level >2.95 mmol/L serving as a predictor of 30-day mortality (AUC 0.805). Similarly, the current study confirmed the significant predictive value of 24 h postoperative Lac for short-term mortality of TAAD patients (AUC 0.688). Moreover, the 72 h postoperative Lac level showed superior predictive efficacy (AUC 0.838) compared to the 24 h value, suggesting that appropriate extension of the monitoring window can better capture the trajectory of metabolic disturbances associated with mortality risk. Based on the aforementioned findings, it can be inferred that Lac may represent a viable therapeutic target for clinical management. In clinical practice, if the Lac level fails to reach the target threshold (Lac ≤7.85 mmol/L) within the first 24 h of treatment initiation, more aggressive interventions are warranted. These strategies may encompass enhanced circulatory support, optimized volume management, and a thorough investigation for potential occult malperfusion or infection. It is important to reduce Lac to the target level of ≤1.75 mmol/L within 72 h; otherwise, patients face a significantly elevated risk of mortality.

In contrast to absolute Lac levels, LC represents the relative rate of change in Lac level over time. Reduced LC has been associated with multiple organ failure and increased mortality in studies of sepsis, trauma, and respiratory failure [14,20]. Notably, survivors of cardiogenic shock exhibited significantly higher LC at 6–8 h and at 24 h after treatment, indicating that LC is an important prognostic indicator (AUC 0.791) [14]. Wang et al. [21] found that elevated Lac at ICU admission (OR 1.284) and decreased LC at 24 h (OR 0.237) were independent risk factors for 30-day mortality in TAAD patients receiving continuous renal replacement therapy (CRRT). Despite the relatively limited research supporting LC as a specific therapeutic target in the context of postoperative TAAD, the present study demonstrates that LC has significant independent prognostic value. The moderate AUC for LC is probably attributable to individual factors, including perioperative hemodynamic stability, organ perfusion status, and the timing of peak Lac levels. This finding highlights the importance of LC as a dynamic metabolic indicator for clinical risk stratification in surgically treated TAAD patients. Furthermore, our results indicate the trend for LC in predicting short-term mortality risk in TAAD is consistent with that of absolute Lac levels, with both demonstrating optimal predictive performance at 72 h post-surgery (AUC 0.667).

Accumulating evidence indicates that elevated Lac levels and reduced LC are significantly associated with adverse clinical outcomes [1,11,15,16]. However, most studies to date have focused on the preoperative period, or within 24 h. The present study confirmed that Lac and LC cut-off values on postoperative day 3 can effectively stratify patient prognosis. Patients had a significantly increased risk of short-term mortality when Lac was >1.75 mmol/L or LC was ≤37.23%. This underscores the potential clinical value of both Lac and LC as prognostic biomarkers for TAAD. The Lac cut-off value identified in this study was relatively lower compared to previous studies, possibly due to the longer observation period of up to 72 h post-surgery. A novel finding of this work was the importance of the dynamic trajectory of Lac within the first 72 h after TAAD surgery, shifting the focus of postoperative management from reliance on static measurements toward a more nuanced, trend-based systemic assessment. By monitoring the Lac trajectory and calculating LC, physicians can accurately identify high-risk patients. For these individuals, timely implementation of more intensive monitoring and aggressive intervention is warranted. Conversely, overtreatment can be avoided in low-risk patients.

During circulatory dysfunction, the patient’s metabolic status directly affects the Lac clearance process. Lac is primarily metabolized by the liver and, to a lesser extent, by the kidneys [22,23]. When liver or kidney function is impaired, the efficiency of Lac clearance decreases, which can lead to a relative increase in Lac levels. Additionally, factors such as age and sex also influence the basal metabolic rate of Lac [6,7]. The current study showed that age, sex, liver function, and kidney function did not significantly affect the predictive value of Lac and its clearance rate for the risk of short-term mortality in TAAD surgical patients, providing indirect support for the robustness of these indicators. Further analysis revealed that Lac had a stronger predictive value for short-term mortality among patients who were older and had poorer liver function (ALT) but relatively preserved kidney function (Cr). Moreover, the protective association demonstrated by LC was also significant. While the predictive value of Lac showed no significant difference between the sexes, the predictive value of LC was stronger in male patients. However, no significant interactions were observed between age, sex, renal function, or liver function and the predictive value of Lac or LC for patient outcome (all *p* for interaction > 0.05). This aligns with the results of a previous study [15].

## 5. Limitations

Several limitations of this research should be acknowledged. First, all patient data for this single-center retrospective study were extracted from an electronic medical record system. The sample size was relatively limited, and cases with extensive missing data were excluded, which may have introduced selection bias. Consequently, the findings may not be fully generalizable to the broader TAAD population. Second, the lack of significant interactions among age, sex, Cr, ALT, and either Lac or LC in the subgroup analysis may be attributed to several factors, including the limited sample size, the relative homogeneity of the study population, and the sensitivity of biomarkers employed to evaluate hepatic and renal injury. Furthermore, CRRT may exert both direct and indirect effects on circulating Lac levels and LC; however, due to constraints in data availability, this variable was not incorporated into the current analysis. Future research with larger sample sizes, more sensitive biomarkers, and the inclusion of critical variables such as CRRT is necessary to further validate the prognostic significance of Lac and LC. Finally, this study analyzed Lac levels only at three fixed time points, failing to capture the complete trajectory of Lac changes. Future research should undertake a more comprehensive investigation of the temporal changes in Lac levels.

## 6. Conclusions

In conclusion, the dynamic changes in Lac levels following surgery in patients with TAAD are closely linked to adverse clinical outcomes. Notably, the Lac level on the third postoperative day, along with its clearance rate, serves as a reliable and effective predictor of short-term mortality. Continuous monitoring of Lac levels post-TAAD surgery provides an early warning of poor prognosis, thereby shifting the focus of precision management based on the dynamic trends of these indicators. In clinical practice, Lac and its clearance may also represent potential therapeutic targets to improve postoperative care in high-risk patients. Furthermore, they could facilitate the implementation of individualized and precise treatment strategies, ultimately reducing short-term mortality.

## Data Availability

The datasets used and analyzed during the current study are available from the corresponding author on reasonable request.

## Abbreviations

TAAD, Type A Aortic Dissection; TBAD, Type B Aortic Dissection; HR, Heart Rate; SBP, Systolic Blood Pressure; DBP, Diastolic Blood Pressure; BMI, Body Mass Index; COPD, Chronic Obstructive Pulmonary Disease; Symptom-to-Surgery Time, Time from Symptom Onset to Surgery; TEVAR, Thoracic Endovascular Aortic Repair (for descending aorta); CABG, Coronary Artery Bypass Grafting; Temporary pacing, Temporary Pacemaker Implantation; PCI, Percutaneous Coronary Intervention; CPB Time, Cardiopulmonary Bypass Time; ACC Time, Aortic Cross-Clamp Time; DHCA, Deep Hypothermic Circulatory Arrest; ICU LOS, Intensive Care Unit Length Of Stay; MV Time, Mechanical Ventilation Time; CRRT, Continuous Renal Replacement Therapy; MODS, Multiple Organ Dysfunction Syndrome; AD, Aortic Diameter; LATD, LA Transverse Diameter; IVSd, Interventricular Septum Thickness at end-diastole; LVPWd, Left Ventricular Posterior Wall thickness at end-diastole; LVIDs, Left Ventricular Internal Dimension at end-Systole; LVEDV, Left Ventricular End-Diastolic Volume; LVESV, Left Ventricular End-Systolic Volume; SV, Stroke Volume; LVFS, Left Ventricular Fractional Shortening; LVEF, Left Ventricular Ejection Fraction; MV E/A, Mitral Valve E/A; MR Vmax, Mitral Regurgitation Peak Velocity; AV Vmax, Aortic Valve Peak Systolic Velocity; WBC, White Blood Cell; NEUT%, Neutrophils Percentage; RBC, Red Blood Cell; PLT, Platelet; PT, Prothrombin Time; Cr, Creatinine; UA, Uric Acid; BG, Blood Glucose; ALT, Alanine Aminotransferase; AST, Aspartate Aminotransferase; AST/ALT, AST to ALT Ratio; TBIL, Total Bilirubin; DBIL, Direct Bilirubin; IBIL, Indirect Bilirubin; TP, Total Protein; α-HBDH, α-Hydroxybutyrate Dehydrogenase; CK, Creatine Kinase; CKMB, Creatine Kinase-MB; TnI, Troponin I; Lac, Lactate; LC, Lactate Clearance; IPO, Immediately Postoperative; POD1, Postoperative Day 1; POD3, Postoperative Day 3.
