# Supplementary material for: Dynamic Lactate Level: An Effective Predictor of Short-Term Mortality After Type A Aortic Dissection Surgery
Source: Rev Cardiovasc Med. 2026 Jun 29;27(6):50931. doi: 10.31083/RCM50931 (PMC13339224; doi:10.31083/RCM50931)
Supplement: Supplementary file 1 [file 2153-8174-27-6-50931-s1.zip › Supplementary Material.pdf]

Dynamic Lactate Level: An Effective Predictor of Short-Term Mortality After Type A Aortic Dissection Surgery

Supplementary Materials

Supplemental Table 1. Multivariable time-dependent Cox regression adjusted for myocardial infarction (MI)

Variable	HR	95% CI	*p*
Lac	1.30	(1.16-1.45)	<0.001
α-HBDH	1.00	(1.00-1.00)	0.545
PLT	0.99	(0.98-1.00)	0.018
Cr	1.00	(1.00-1.01)	0.138
D-dimer	1.00	(1.00-1.00)	0.096
PT	0.96	(0.86-1.07)	0.472
TnI	1.02	(0.97-1.08)	0.453
LATD	1.17	(1.01-1.36)	0.040
AST/ALT	1.03	(1.00-1.05)	0.034
CPB Time	1.00	(0.98-1.01)	0.351
CABG	1.01	(0.36-2.84)	0.981
Surgery Duration	1.47	(1.12-1.93)	0.005
Age	1.06	(1.02-1.10)	0.007
Bentall Procedure	2.51	(0.95-6.66)	0.064
MV Time	1.00	(1.00-1.01)	0.139
Temporary Pacing	0.53	(0.25-1.12)	0.097
MI	1.69	(0.60-4.72)	0.319

HR, hazard ratio; CI, confidence interval; Lac, Lactate; α-HBDH, α-Hydroxybutyrate Dehydrogenase; PLT, Platelet; Cr, Creatinine; PT, Prothrombin Time; TnI, Troponin I; LATD, LA Transverse Diameter; ALT, Alanine Aminotransferase; AST, Aspartate Aminotransferase; AST/ALT, AST to ALT Ratio; CPB Time, Cardiopulmonary Bypass Time; CABG, Coronary Artery Bypass Grafting; Temporary pacing, Temporary Pacemaker Implantation; MV Time, Mechanical Ventilation Time.

Supplemental Table 2. Multivariable time-dependent Cox regression in patients without MI

Variable	HR	95% CI	*p*
Lac	1.50	(1.19-1.90)	0.001
α-HBDH	1.00	(1.00-1.00)	0.133
PLT	0.99	(0.97-1.00)	0.132
Cr	1.01	(1.00-1.02)	0.061
D-dimer	1.00	(1.00-1.00)	0.012
PT	1.08	(0.92-1.28)	0.355
TnI	0.97	(0.87-1.07)	0.512
LATD	1.24	(0.99-1.55)	0.060
AST/ALT	0.72	(0.28-1.86)	0.499
CPB Time	1.00	(0.99-1.02)	0.579
CABG	1.62	(0.12-21.52)	0.713
Surgery Duration	1.09	(1.02-1.17)	0.017
Age	1.86	(1.24-2.78)	0.003
Bentall Procedure	1.00	(1.00-1.01)	0.468
MV Time	0.59	(0.16-2.15)	0.428
Temporary Pacing	2.08	(0.35-12.21)	0.418

Supplemental Table 3. Tests of Model Effects in Generalized Estimating Equations (GEE)

Effect	Wald χ^2	df	*p*
(Intercept)	26.346	1	<0.001
Lac	4.209	1	0.040
α-HBDH	5.115	1	0.024
PLT	3.439	1	0.064
Cr	0.302	1	0.583
D-dimer	0.508	1	0.476
PT	4.839	1	0.028
TnI	2.080	1	0.149
LATD	0.286	1	0.593
AST/ALT	1.123	1	0.289
CPB Time	4.653	1	0.031
CABG	2.764	1	0.096
Surgery Duration	9.701	1	0.002
Age	6.414	1	0.011
Bentall Procedure	0.247	1	0.619
MV Time	8.912	1	0.003
Temporary Pacing	3.759	1	0.053

Supplemental Table 4. GEE Analysis for Prognostic Factors in TAAD Patients Undergoing Surgery

Variable	OR	95% CI	*p*
Lac	1.52	(1.02-2.25)	0.040
α-HBDH	1.00	(1.00-1.00)	0.024
PLT	1.01	(1.00-1.01)	0.064
Cr	1.00	(0.99-1.01)	0.583
D-dimer	1.01	(0.98-1.04)	0.476
PT	1.16	(1.02-1.32)	0.028
TnI	1.04	(0.99-1.10)	0.149
LATD	1.03	(0.92-1.17)	0.593
AST/ALT	1.44	(0.73-2.82)	0.289
CPB Time	1.01	(1.00-1.03)	0.031
CABG	4.05	(0.78-21.08)	0.096
Surgery Duration	2.13	(1.32-3.43)	0.002
Age	1.07	(1.02-1.13)	0.011
Bentall Procedure	1.43	(0.35-5.93)	0.619
MV Time	1.02	(1.01-1.03)	0.003
Temporary Pacing	3.10	(0.99-9.73)	0.053

OR, odds ratio.
